# Role of Tyrosine Phosphorylation in PEP1 Receptor 1(PEPR1) in *Arabidopsis thaliana*

**DOI:** 10.3390/plants14101515

**Published:** 2025-05-19

**Authors:** Jae-Han Choi, Man-Ho Oh

**Affiliations:** Department of Biological Sciences, College of Biological Sciences and Biotechnology, Chungnam National University, Daejeon 34134, Republic of Korea; jaehan961@naver.com

**Keywords:** leucine-rich repeat receptor-like kinases, *PEPR1* and *PEPR2*, pep1 peptide, tyrosine phosphorylation, *Arabidopsis thaliana*

## Abstract

Leucine-rich repeat receptor-like kinases (LRR-RLKs) have evolved to perceive environmental changes. Among LRR-RLKs, PEPR1 perceives the pep1 peptide and triggers defense signal transduction in *Arabidopsis thaliana*. In the present study, we focused on PEPR1 and PEPR2, which are the receptors of pep1, to understand the role of tyrosine phosphorylation. PEPR1-CD (cytoplasmic domain) recombinant protein exhibited strong tyrosine autophosphorylation, including threonine autophosphorylation. We subjected all tyrosine residues in PEPR1-CD to site-directed mutagenesis. The recombinant proteins were purified along with PEPR1-CD, and Western blotting was performed using a tyrosine-specific antibody. Among the 13 tyrosine residues in PEPR1-CD, the PEPR1(Y995F)-CD recombinant protein showed significantly reduced tyrosine autophosphorylation intensity compared to PEPR1-CD and other tyrosine mutants, despite little change in threonine autophosphorylation. To confirm the autophosphorylation site, we generated a phospho-specific peptide Ab, pY995. As a result, Tyr-995 of PEPR1-CD was a major tyrosine autophosphorylation site in vitro. To understand the function of tyrosine phosphorylation in vivo, we generated transgenic plants, expressing PEPR1-Flag, PEPR1(Y995F)-Flag, and PEPR1(Y995D)-Flag in a *pepr1/2* double mutant background. Interestingly, the root growths of PEPR1(Y995F)-Flag and PEPR1(Y995D)-Flag were not inhibited by pep1 peptide treatment, compared to Col-0 and PEPR1-Flag (*pepr1/2*) transgenic plants. Also, we analyzed downstream components, which included PROPEP1, MPK3, WRKY33, and RBOHD gene expressions in four different genotypes (Col-0, PEPR1-Flag, PEPR1(Y995F)-Flag, and PEPR1(Y995D)-Flag) of plants in the presence of the pep1 peptide. Interestingly, the expressions of PROPEP1, MPK3, WRKY33, and RBOHD were not regulated by pep1 peptide treatment in PEPR1(Y995F)-Flag and PEPR1(Y995D)-Flag transgenic plants, in contrast to Col-0 and PEPR1-Flag. These results suggest that specific tyrosine residues play an important role in vivo in the plant receptor function.

## 1. Introduction

Plants cannot avoid various stress situations, such as attacks by pathogens, unlike animals. Therefore, they have evolved to have complex systemic defense mechanisms. For example, pattern recognition receptors (PRRs), such as FLAGELLIN-SENSING2 (FLS2), EF-Tu receptor (EFR), PEP RECEPTOR1 (PEPR1), and ELICITOR RECEPTOR KINASE 1 (CERK1), that recognize specific ligands have been reported [1,2]. Among PRRs, receptors with a domain in which leucine is repeated in the extracellular domain are classified as leucine-rich repeat receptor-like kinases (LRR-RLKs), which form a large gene family in *Arabidopsis thaliana* [3]. In particular, FLS2 recognizes a specific amino acid sequence of bacterial flagellin (flg22); EFR recognizes an elongation factor (elf18); CERK1 recognizes chitin, a component of the cell wall of fungi; and plant PEPR1 recognizes the plant elicitor peptide (pep1) [4,5,6,7,8,9,10,11]. Pep1, a signaling peptide consisting of 23 amino acids, which is ATKVKAKQRGKEKVSSGRPGQHN [12], is the ligand recognized by PEPR1 and PEPR2 [13,14]. Pep1 is derived from its precursor, PROPEP1, and activates the plant’s innate immune response through the expression of plant defensin (PDF), which encodes the defensin family, and pathogenesis-related protein-1 (PR-1) [2,12]. According to the trade-off concept between plant growth and defense, when plants are attacked by pathogens, plant growth is inhibited. In particular, the growth of the primary and lateral roots almost stops, and the formation of root hair is also inhibited [15,16,17,18]. Similarly, plant innate defense signaling of the PEPR1–pep1 complex also inhibits root growth by generating ROS through interaction with the RBOHD gene as part of the plant defense response [19]. 

In terms of intracellular signal transduction in plants, post-translational modifications (PTMs) are modifications that occur after proteins are translated and affect the structure, activity, location, and function of proteins. This process is reversible and acts as a regulator of various metabolisms [20]. More than 300 types of PTMs are known, including phosphorylation, acylation, methylation, SUMOylation, and ubiquitination. Among them, phosphorylation occurs frequently enough to account for 1/3 of the entire PTM process and plays an important role in various signaling pathways within cells. In plants, phosphorylation mainly occurs in serine and threonine amino residues, but phosphorylation of tyrosine is also observed with considerable frequency [20,21,22,23,24,25]. Tyrosine phosphorylation has been reported to play a major role in the signaling of BRASSINOSTEROID INSENSITIVE 1 (BRI1) in Arabidopsis [24,26,27,28], and EFR tyrosine phosphorylation and bacterial tyrosine phosphatase with studies on functional inhibition have been reported [29], studies on the tyrosine phosphorylation of protein kinase complexes that function in plant defense systems have also been reported [30]. Therefore, it is thought that the importance of tyrosine phosphorylation in plants can be considered through the study of tyrosine residues, rather than the phosphorylation of threonine and serine residues. In the present study, to understand the function of PEPR1 tyrosine phosphorylation in vivo, we identified a major tyrosine autophosphorylation site and generated transgenic plants, which included PEPR1-Flag, PEPR1(Y995F)-Flag, and PEPR1(Y995D)-Flag, in a *pepr1/2* double mutant background. Root growth was tested in half-strength MS media with/without pep1, and gene expressions of downstream components in four different genotypes, Col-0, PEPR1-Flag, PEPR1(Y995F)-Flag, and PEPR1(Y995D)-Flag, were analyzed.

## 2. Materials and Methods

### 2.1. Site-Directed Mutagenesis (SDM)

Primers of Site-directed mutagenesis (SDM) were designed to substitute tyrosine residues with phenylalanine in the PEPR1-CD and PEPR1 full-length coding sequences and PEPR2-CD cloned into the pFlag-Mac vector, which generated SDM with specific SDM primers for Y805F, Y831F, Y842F, Y852F, Y901F, Y903F, Y910F, Y929F, Y941F, Y944F, Y995F, Y1013F, and Y1015F and PEPR2 SDM primers Y798F, Y809F, Y819F, Y868F, Y870F, Y908F, Y962F, Y969F, Y980F, Y982F, and Y1017F, as well as pBIB-Hyg^+^-35S-PEPR1-Flag, pBIB-Hyg^+^-35S-PEPR1(Y995F)-Flag, and pBIB-Hyg^+^-35S-PEPR1(Y995D)-Flag.

Using the designed primer, PEPR1- and PEPR2-cloned plasmid DNA, and pfu DNA polymerase (Cat. No. PD116-500, BioFact™, Daejeon, Republic of Korea), the experiment was performed at 95 °C for 30 s/95 °C for 30 s, 55 °C for 1 min, and 72 °C for 8 min/x20 cycles (2 μL pfu, 50ng plasmid DNA, 10 pmol/μL primers, each 10 mM dNTPs, 10X buffer). Thereafter, in order to verify the base sequence, it was sequenced in both directions and confirmed [24]. 

### 2.2. Recombinant Protein Production and Purification

To produce recombinant proteins, *E. coli* BL21 (DE3) (Novagen, Temecula, CA, USA) cells were transformed with vectors containing the genes of interest. *E. coli* cells were grown in Luria–Bertani (LB) medium, and the expressions of Flag-PEPR1-CD, including SDM mutants of tyrosine residues, and Flag-PEPR2-CD were induced with 0.3 mM isopropyl β-D-1-thiogalactopyranoside (IPTG) (Sigma-Aldrich, St. Louis, MO, USA) when the optical density (OD600) of the cell culture reached 0.6. For maximum recombinant protein production, *E. coli* cells were incubated at room temperature with shaking for 16 h following IPTG induction. Cells were harvested by centrifugation and resuspended in a buffer containing 50 mM 3-(N-morpholino) propanesulfonic acid (MOPS) (pH 7.5), 150 mM NaCl, and protease inhibitors before being lysed by sonication. Cell lysates were fractionated by centrifugation at 35,000× *g* into soluble and pellet fractions. The recombinant FLAG-tagged proteins in the soluble fractions were immunopurified on an anti-FLAG M2 affinity gel (Sigma-Aldrich, St. Louis, MO, USA).

After purification, the protein solutions were dialyzed against a 1000× volume of dialysis buffer containing 20 mM MOPS (pH 7.5) and 1 mM dithiothreitol (DTT) (Sigma-Aldrich, St Louis, MO, USA), as previously described [26]. After the wash was completed, 300 μL of elution buffer (final 50 mM MOPS, pH 7.5, 150 mM NaCl, 100 μg/mL FLAG peptide) was added and shaken at 4° C for 30 min for elution. Then, the sample was placed into SnakeSkin™ dialysis tubing (Cat. No. 68305, Thermofisher Scientific Inc., Waltham, MA, USA), both inlets were sealed, it was placed in a dialysis buffer (final 20 mM MOPS, pH 7.5, 1 mM DTT), and stirred at 4 °C. It was shaken for 4 h on the machine [24]. 

### 2.3. Electrophoresis and Immunoblotting

Recombinant protein preparations were mixed with pre-heated (95 °C) 1× sodium dodecyl sulfate polyacrylamide gel electrophoresis (SDS-PAGE) sample buffer containing 1 M urea, 0.7 M 2-mercaptoethanol, 5 mM NaF, 1 mM Na2MoO4, 1 mM Na_3_VO_4_, 1 mM aminoethylbenzenesulfonyl fluoride, and 2 mM EDTA. Protein concentrations were determined by the dye-binding assay (Bio-Rad, Hercules, CA, USA) using bovine serum albumin as a protein standard. Proteins were separated on 12% polyacrylamide (0.1% SDS) gels and transferred to polyvinylidene difluoride (PVDF) fluorescence-specific membranes (Millipore, Bedford, MA, USA). Membranes were blocked in a 2% (*v*/*v*) fish gelatin solution in phosphate-buffered saline (PBS; 5 mM NaH2PO4, 150 mM NaCl, pH 7.4) before being incubated with primary antibodies, which were diluted as specified in PBS with containing 0.1% (*v*/*v*) Tween-20 (PBST). Purified recombinant proteins or proteins in the soluble fraction were analyzed using SDS-PAGE and immunoblotting with anti-FLAG antibodies (1:5000 dilution, Sigma–Aldrich), antiphosphothreonine antibodies (1:500 dilution, Invitrogen, Carlsbad, CA, USA), and antiphosphotyrosine antibodies (1:500 dilution, Invitrogen, CA, USA) to monitor the overall pattern of phosphorylation and level of recombinant protein expression in *E. coli*. custom modification antibodies (anti-pY831, anti-pY956, or anti-pY1072 antibodies). The custom antibodies were produced against the following sequences: pY995 and VTGTTGpYIAPENAF. The antibody was produced by GenScript and sequentially affinity-purified by using the nonphosphorylated and phosphotyrosine-containing antigen peptides.

Immunoblots were scanned using an Odyssey C-Digit scanner (LI-COR Bioscience, Lincoln, NE, USA) for visualization.

### 2.4. Seedling Growth and Root Length Measurement

Seeds were surface-sterilized using 70% ethanol for 1 min and 50% sodium hypochlorite for 8 min, followed by washing with sterilized water. After 2 days of stratification at 4 °C, the seeds were plated on half-strength Murashige and Skoog (MS) basal salt medium containing 1% sucrose (pH 5.7) and 0.8% agar in the presence of the pep1 peptide (1 μM), with control in a controlled growth chamber, with 130 µmol photons (PAR) m^−2^ s^−1^ and a 16 h light/8 h dark cycle at 22 °C. After 7 days, root growths of seedlings were photographed, and root lengths were measured Half of the 1/2 MS medium was treated with pep1 (1 μM), and the other half was not treated on the solid agar medium as a control. Col-0, pepr1/2 double knock-out plants, and transgenic plants were planted in each medium and grown vertically for 10 days. Afterwards, photos of the plants were taken, and the lengths of the primary roots of 60 plants were measured using the image J program.

### 2.5. Liquid Culture and Ligand Treatment

Seeds were surface-sterilized, stratified for 2 days at 4 °C in the dark, and cultured in half-strength liquid MS media under continuous light. The 11-day-old seedlings were cultured once more after changing into the same fresh media [24]. In order to treat the cultured seedlings under −/+ pep1, half of the samples were not treated with the ligand, and the other half of the samples were treated with pep1 (1 μM) at 23 °C for 2 h.

### 2.6. Total RNA Extraction and cDNA Synthesis

Harvested samples were ground using a pestle and mortar filled with sterilized liquid nitrogen. Then, total RNA was extracted using the EZ Total RNA miniprep Kit (Cat. No. EP301-50N, ENZYNOMICS, Daejeon, Republic of Korea). β-mercaptoethanol was added to Buffer ELB to create a solution, and the ground sample was added to 700 μL of the solution, vortexed, and incubated at room temperature for 3 min. The solution was placed into a gDNA-elimination spin column and centrifuged (13,000 rpm, 10 min), and 700 μL of 70% EtOH with DEPC-treated water was added to the solution. Thereafter, the mixed solution was transferred to an RNA mini spin column and centrifuged (13,000 rpm, 1 min), and the solution passing through the column was discarded and treated with DNaseI. It reacted at room temperature for 5 min, and RNA was extracted through washing and elution processes.

cDNA was synthesized using 1 μg of total RNA and ReverTra Ace -α- (Cat. No.FSK-101, TOYOBO, Osaka, Japan). We added 1 μg of total RNA + RNase free water = 10 μL and ReverTra Ace 1 μL + RNase inhibitor 1 μL + each 10 mM dNTPs mixture 2 μL + 10 pmol/μL Oligo(dT)20 2 μL + 5X buffer (contains 25 mM Mg^2+^) 4 μL = 10 μL for a total of 20 μL. Elongation was performed at 42 °C for 20 min, and enzyme inactivation was performed at 99 °C for 5 min [31].

### 2.7. Quantitative Real-Time Polymerase Chain Reaction (qRT-PCR) and Statistical Analysis

PCR was performed using 1 μL of cDNA, 2 μL of 10 pmol/μL qRT-PCR primers, 2 μL of H_2_O, TOPreal™ qPCR 2X PreMIX (SYBR Green with low ROX) (Cat. No. RT5005, ENZYNOMICS, Daejeon, South Korea), 5 μL each, using the CFX Connect Real-Time PCR system (Cat. No. BR1855200, BIO-RAD, Hercules, CA, USA) equipment. Denaturation was performed at 95 °C for 15 min/95 °C for 10 s; annealing was performed at 60 °C for 15 s; elongation was performed at 72 °C for 30 s/x 45 cycles and for 5 s from 55 °C to 95 °C, measuring the melting curve at 0.5 °C intervals. The Δcq value of each gene was normalized against the Δcq value of ACTIN, and the ΔΔcq value was obtained to analyze the expression level. Then, the significance of the result was analyzed using a *t*-test [31]. 

### 2.8. Agrobacterium-Mediated Transformation into pepr1/2 Double Knock-Out Arabidopsis Plants

For plant transformation, PEPR1, PEPR1(Y995F)-Flag, and PEPR1(Y995D)-Flag plasmid DNA inserted into the JJ461 vector were transformed into an Agrobacterium cell line (GV3101). The cells were plated on solid LB medium supplemented with kanamycin (50 μg/mL) and rifampicin (10 μg/mL) and incubated at 28 °C for 48 h. The colony was inoculated into liquid LB medium supplemented with kanamycin (50 μg/mL) and rifampicin (10 μg/mL) and cultured. After harvesting the cells by centrifugation (23 °C, 10 min), the cells were resuspended using a 5% sucrose solution, and silwet L-77 (final 0.02%) was added. Then, *pepr1/2* double knock-out plants grown for 4 weeks at 23 °C were immersed in the solution for 15 s, sealed with vinyl, and grown in dark conditions for one day, and then the vinyl was removed [32]. 

### 2.9. Selection of Transgenic Plants

After surface sterilization of the harvested seeds, the seeds obtained from the transformed plants were seeded in 1/2 MS medium supplemented with hygromycin-B (25 μg/mL) for selection. Selected individuals were transplanted in soil. After harvesting the seeds, selection was repeated through three generations to select 3 different homozygous lines. After selection, total membrane proteins were extracted, and the confirmation overexpression of target genes was performed with Western blot using Flag Ab.

### 2.10. Genes Cloning

For the cloning of the gene used in this study, the cDNA of Arabidopsis was used to amplify the DNA through PCR using pfu DNA polymerase (Cat. No. PD116-250, BioFact™, Daejeon, Republic of Korea), and the pENTR™/D-TOPO™ cloning vector (Cat. No. K240020, Thermofisher Scientific Inc., Waltham, MA, USA) was used as an entry vector and ligated for 1 h at room temperature. After ligation, DH5α-competent cells were transformed, plated on LB medium supplemented with kanamycin (50 μg/mL), and cultured at 37 °C for 16 h. After primary confirmation through colony PCR, plasmid DNA was extracted by mini prep, and sequencing was requested to Cosmogenetech to confirm the sequence. Then, using 50 ng of plasmid DNA, 150 ng of destination vector, and Gateway LR clonase (Cat. No. 11791020, Thermofisher Scientific Inc., MA, USA), the reaction was performed at 25 °C for 4 h, followed by transformation into DH5α-competent cells. After extracting plasmid DNA, it was transformed into the BL21(DE3) or the GV3101 cell line according to the purpose of each experiment.

### 2.11. BiFC Assay

Reverse reaction (LR) recombinations of appropriate open reading frames (AtBIK1, At3g07070, and At4g02630) in pENTR/D-TOPO were performed with the split-YFP destination vectors pDEST-GWVYNE and pDEST-GWVYCE to generate N- or C-terminal fusions with the N- and C-terminal yellow fluorescent protein (YFP) moieties, respectively [33,34]. Recombined vectors were transformed into the Agrobacterium strain GV3101. Six-week-old *Nicotiana benthamiana* leaves were agro-filtrated, as previously described [35]. After 48 h, YFP fluorescence was visualized using a super-resolution confocal laser scanning microscope (LSM 880 with Airyscan, Zeiss, Jena, Germany).

## 3. Results

### 3.1. Identification of Major Tyrosine Autophosphorylation Site of PEPR1-CD and PEPR2-CD

**Figure 1:**
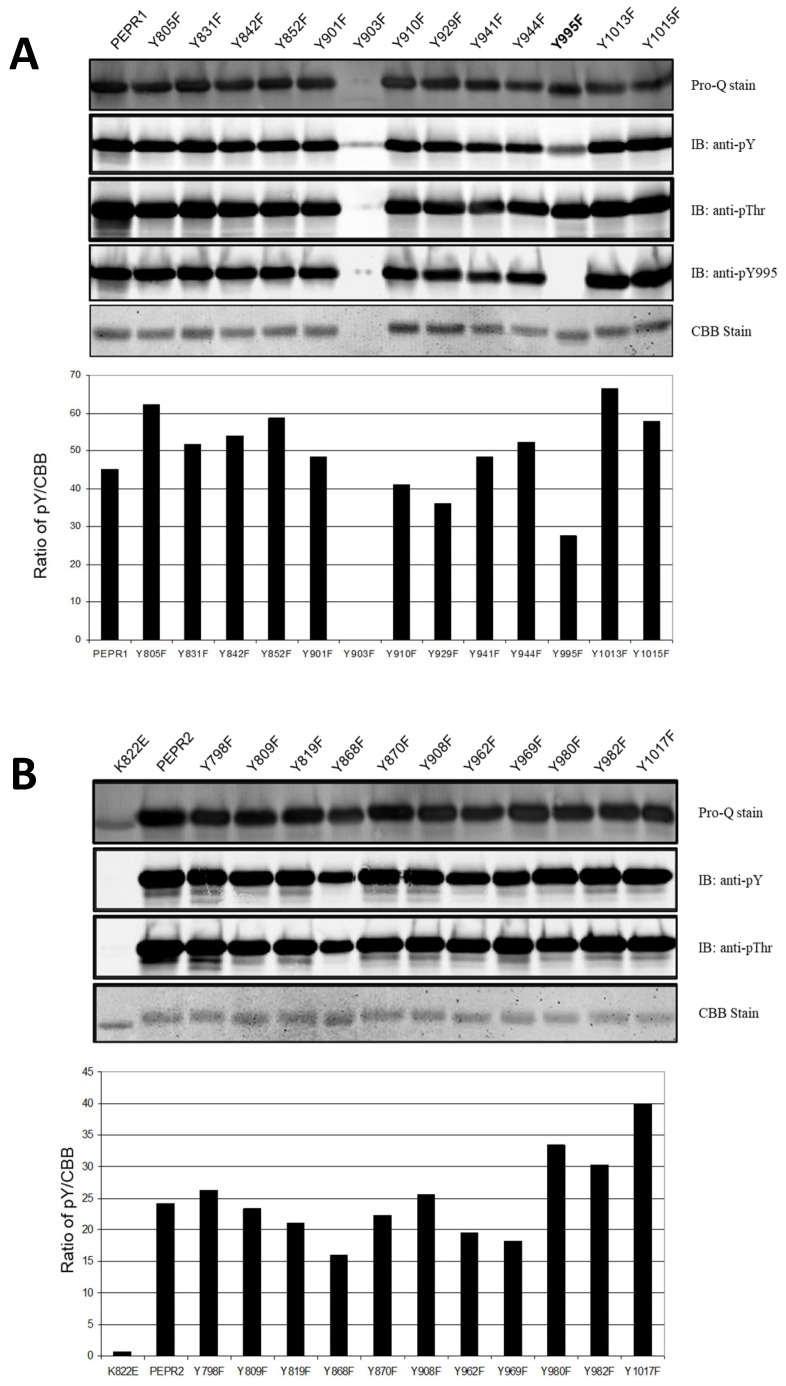
Autophosphorylation of the recombinant proteins Flag-PEPR1 and Flag-PEPR2 and Tyr-995 of Flag-PEPR1-CD are major tyrosine autophosphorylation sites in vitro. Recombinant protein was expressed with the pFlag-Mac vector and extracted from an *E. coli* cell and purified. Detection of the autophosphorylation level was performed with pY, pThr, and pY995 antibodies and analyzed using a ratio of pY/CBB. (**A**) Immunoblot analysis was performed with pY, pThr, and pY995 antibodies. (**B**) Immunoblot analysis was performed with pY and pThr antibodies.

In the present study, we identified a major site of tyrosine autophosphorylation. To identify tyrosine phosphorylation site(s), we cloned PEPR1-CD and PEPR2-CD into the pFlag-Mac protein expression vector and subjected all tyrosine residues in Flag-PEPR1-CD and Flag-PEPR2-CD to site-directed mutagenesis. The recombinant proteins were purified along with Flag-PEPR1-CD and Flag-PEPR2-CD, and Western blot analysis was performed using a tyrosine-specific antibody (pY), including a phospho-specific peptide antibody (pY995) and a threonine-specific antibody (pThr). Among the 13 tyrosine residues in Flag-PEPR1-CD, the Flag-PEPR1(Y995F)-CD recombinant protein showed significantly reduced tyrosine autophosphorylation intensity, compared to Flag-PEPR1-CD and other tyrosine mutants, despite little change in threonine autophosphorylation. In the case of Y903F, the recombinant protein was not expressed well. To confirm the autophosphorylation site, we generated phospho-specific peptide Ab, pY995. As a result, we confirmed that the decrease in tyrosine autophosphorylation intensity of Flag-PEPR1(Y995F)-CD was due to the removal of the autophosphorylation in the Tyr-995 residue (Figure 1A). Also, Flag-PEPR2-CD recombinant proteins were purified, and immunoblot analysis was performed using the same method as for Flag-PEPR1-CD. As shown in Figure 1B, among the 12 tyrosine residues in Flag-PEPR2-CD, all recombinant proteins showed no difference in tyrosine autophosphorylation intensity. Flag-PEPR2(K822E)-CD was a kinase-dead recombinant protein, in which the ATP-binding site was substituted with glutamate (E). Therefore, ATP could not bind to Flag-PEPR2(K822E)-CD, resulting in no autophosphorylation kinase activity.

### 3.2. Phenotypic Analysis Under pep1 Treatment with Transgenic Plants

**Figure 2:**
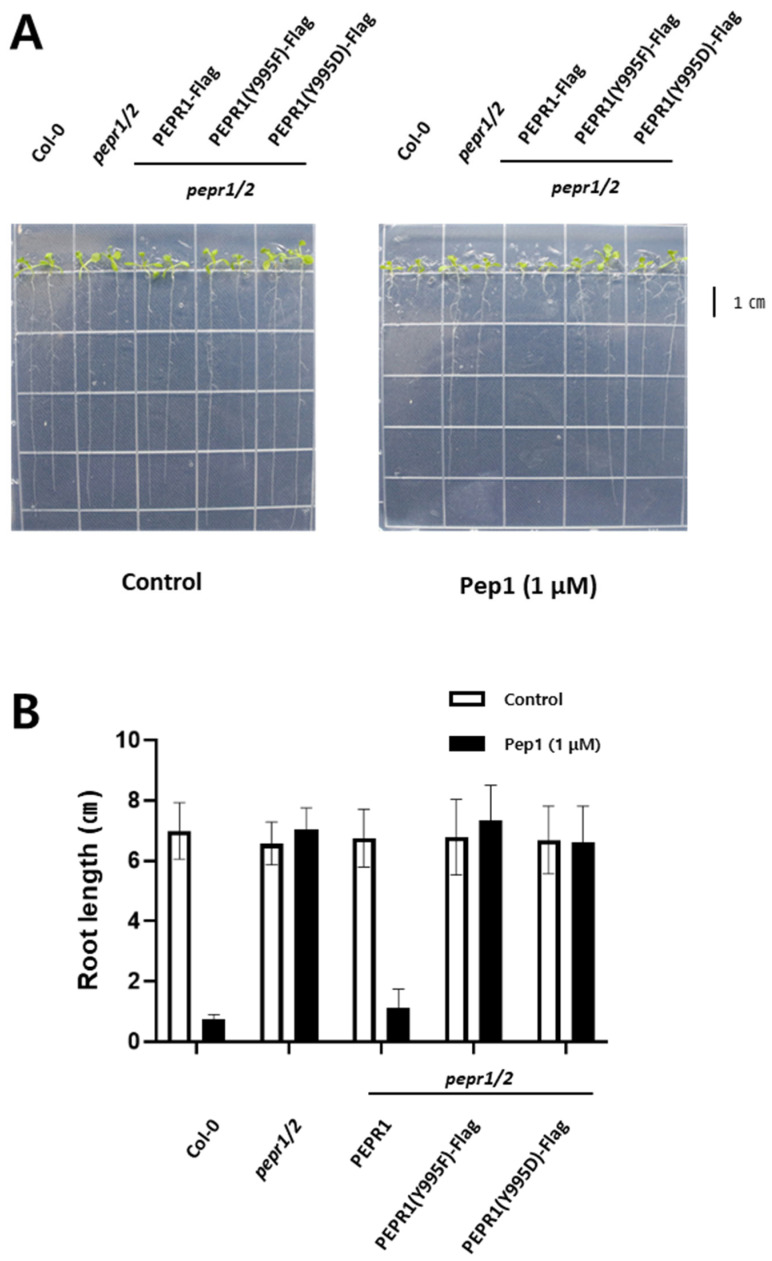
Root growth in Col-0, *pepr1/2* double knock-out, and transgenic plants. PEPR1, PEPR1(Y995F)-Flag, and PEPR1(Y995D)-Flag transformed into *pepr1/2* double knock-out plants through the floral dipping method. (**A**) Root growths of Col-0, *pepr1/2* double knock-out, PEPR1-Flag, PEPR1(Y995F)-Flag, and PEPR1(Y995D)-Flag seedlings in half-strength MS media. (**B**) Primary root lengths of Col-0, PEPR1-Flag, *pepr1/2* double knock-out, PEPR1(Y995F)-Flag, and PEPR1(Y995D)-Flag. Five independent biological repeats were conducted. Error bar represents SE.

Based on Figure 1A, we focused on the pY995 site of PEPR1 in terms of kinase activity and pep1-mediated plant defense signaling. Therefore, we generated transgenic plants using PEPR1-Flag, PEPR1(Y995F)-Flag, and PEPR1(Y995D)-Flag in a *pepr1/2* double knock-out background to understand the functional importance of the Y995 site in vivo. After pep1 treatment, whether the signaling cascade was normally transmitted to downstream components was analyzed based on root growth (Figure 2A,B). The plants of Col-0, *pepr1/2* K.O, PEPR1, PEPR1(Y995F), and PEPR1(Y995D) exhibited the same root growth phenotype in the half-strength MS medium. However, on the pep1-treated medium, Col-0 plants recognized pep1 and initiated defense responses, greatly inhibiting root growth, whereas *pepr1/2* double knock-out plants did not possess a functional receptor to recognize pep1. In the case of PEPR1-Flag (*pepr1/2* background) transgenic plants, they showed the same growth patterns as Col-0 because they were rescued by the normal receptor kinase, which was able to recognize the pep1 peptide in vivo.

In contrast, in PEPR1-Y995F(*pepr1/2*)-Flag and PEPR1-Y995D(*pepr1/2*)-Flag, similar to *pepr1/2* double knock-out plants, root growths were unaffected by pep1 peptide treatment (Figure 2A,B). As shown in Figure 2B, in Col-0 and PEPR1(*pepr1/2*)-Flag grown for 10 days with 1 μM pep1, the primary root was less than 1 cm, and the growth was inhibited more than seven times, compared to the *pepr1/2* double knock-out mutants, PEPR1(Y995F)-Flag and PEPR1(Y995D)-Flag.

### 3.3. Expression Analysis of Downstream Components Involved in pep1 Signaling

**Figure 3:**
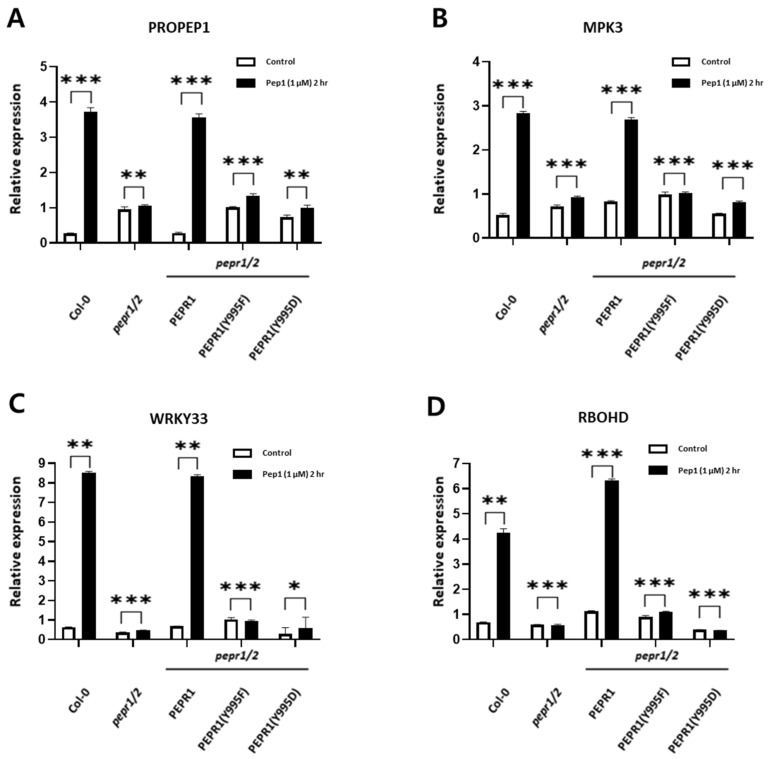
Expression analysis of downstream genes in five genotypes after pep1 treatment. The effects of pep1 on the expression of the pep1 downstream genes in five genotypes. Seedlings of *Arabidopsis* were cultured in half-strength MS medium for 10 days and treated with pep1 peptide for 2 h. PROPEP1, MPK3, WRKY33, and RBOHD expressions were analyzed with pep1 treatment for Col-0, PEPR1-Flag, *pepr1/2* double knock-out mutants, PEPR1(Y995F)-Flag, and PEPR1(Y995D)-Flag. The relative expression was normalized to that of ACTIN. Three independent biological repeats were conducted. Error bar represents SE. * *p* < 0.05, ** *p* < 0.01, and *** *p* < 0.001.

To analyze the expression of downstream genes regulated by a pep1 peptide-triggered signal in COl-0, PEPR1-Flag, PEPR1(Y995F)-Flag, and PEPR1(Y995D)-Flag seedlings, we isolated total RNA, synthesized cDNA, and performed qRT-PCR for four different genes: *PROPEP1*, *MPK3*, *WRKY33*, and *RBOHD*. In this experiment, the expression levels of these four genes were significantly increased in both Col-0 and PEPR1-Flag transgenic seedlings after 2 h of pep1 treatment (Figure 3). However, their expression levels remained unchanged in *pepr1/2* double knock-out mutant seedlings, PEPR1(Y995F)-Flag, and PEPR1(Y995D)-Flag, indicating that the pep1 signal was not transmitted to downstream signaling components.

### 3.4. Analysis of Expression Patterns of RLCKs Under pep1 Treatment

**Figure 4:**
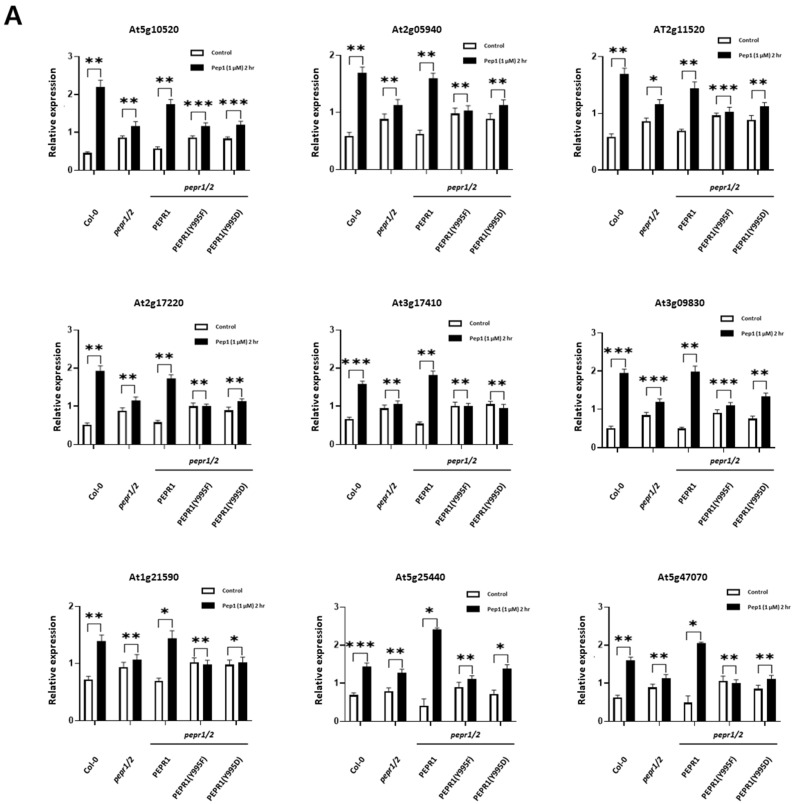

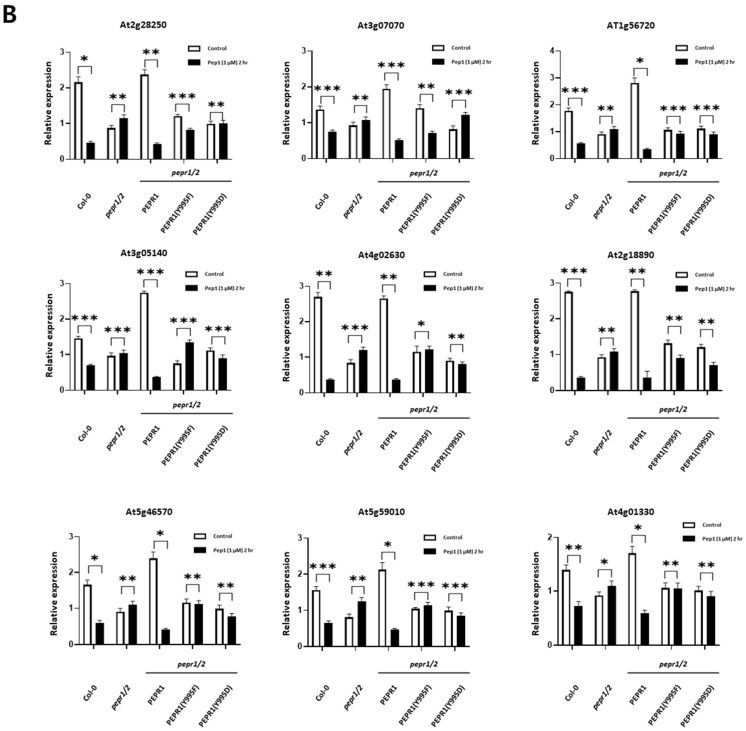
Expression analysis of RLCKs after pep1 treatment in Col-0, *pepr1/2* double knock-out, PEPR1-Flag, PEPR1(Y995F)-Flag, and PEPR1(Y995D)-Flag in *pepr1/2* double mutant background plants. *Arabidopsis* seedlings were cultured in half-strength MS medium for 10 days and treated with pep1 for 2 h. Among 20 RLCKs, 18 RLCKs were regulated by pep1 for Col-0 and PEPR1-Flag, but not for *pepr1/2* K.O, PEPR1(Y995F)-Flag, and PEPR1(Y995D)-Flag in a *pepr1/2* double mutant background. The relative expression was normalized to that of ACTIN. Three independent biological repeats were conducted. Error bar represents SE. * *p* < 0.05, ** *p* < 0.01, and *** *p* < 0.001.

As other interesting signaling components, we focused on the receptor-like cytoplasmic kinases (RLCKs) in pep1-triggered defense signaling as specific RLCKs, except for BIK1, which has not been identified. To identify other RLCKs regulated by pep1 treatment, we also analyzed whether phosphorylation of the Y995 site of PEPR1 affected the expression of RLCK. The expressions of 78 RLCK genes were analyzed after 2 h of pep1 treatment in Col-0. Among them, 11 RLCKs were up-regulated, and 9 RLCKs were down-regulated by pep1 treatment (Supplementary Material Figure S1). Based on these results the expressions of these 20 RLCK genes regulated by pep1 were analyzed in Col-0, *pepr1/2* double knock-out, PEPR1-Flag, PEPR1(Y995F)-Flag, and PEPR1(Y995D)-Flag under the same conditions. As a result, 18 RLCKs were selected. A total of nine RLCKs were up-regulated (Figure 4A), and nine RLCKs were down-regulated (Figure 4B) by pep1 treatment in Col-0 and PEPR1-Flag seedlings. However, these 18 RLCKs were not regulated by pep1 treatment in *pepr1/2* double knock-out, PEPR1-Flag, PEPR1(Y995F)-Flag, and PEPR1(Y995D)-Flag seedlings.

### 3.5. PEPR1 and RLCKs Indirectly Interact Through BIK1

**Figure 5:**
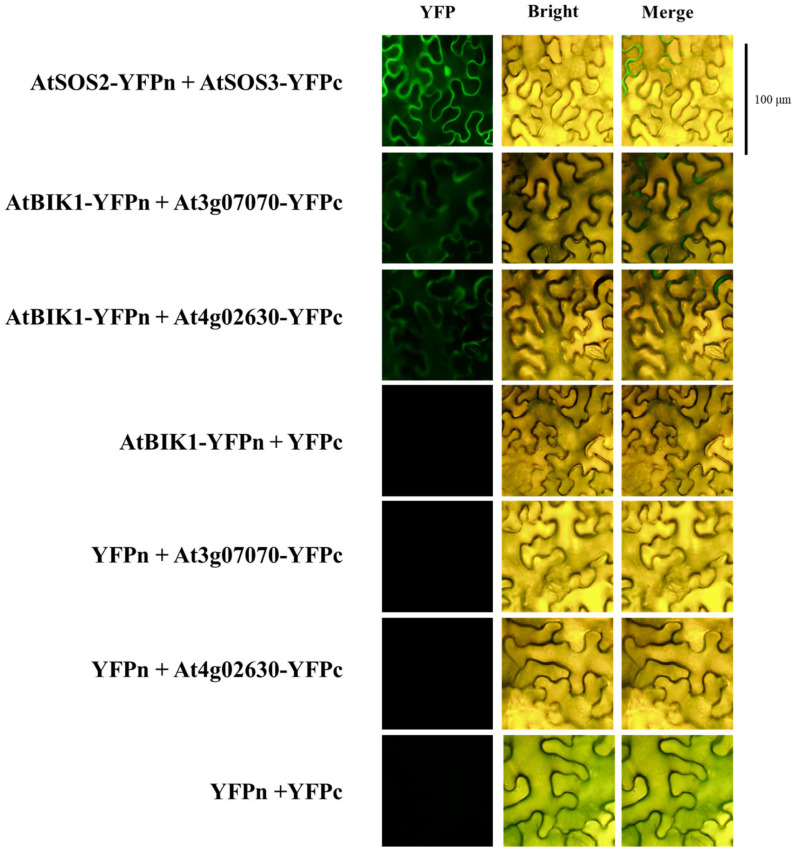
AtBIK1 interacts with At3g07070 and At4g02630 in the plasma membrane. A. BiFC assay demonstrated the interaction between BIK1 and At3g07070. B. BiFC assay demonstrated the interaction between BIK1 and At4g02630 in tobacco (*Nicotiana benthamiana*) leaves. Genes fused with the N-terminal or C-terminal fragment of YFP (YFPn or YFPc) were co-introduced into tobacco leaves. AtSOS2-YFPn and AtSOS3-YFPc were used as positive control. Each empty vector complex was used as the negative control.

We focused on identifying changes in RLCK expression patterns induced by pep1 signaling and determining whether there was a physical interaction. Therefore, a bimolecular fluorescence complementation (BiFC) assay was performed to analyze whether the RLCK proteins interacted directly or indirectly with PEPR1. As a result of BiFC, a direct interaction between PEPR1 and RLCKs tested could not be confirmed. To investigate potential indirect interactions, a BiFC assay was conducted with BIK1, an RLCK involved in defense by interacting with PEPR1. This analysis confirmed that two RLCKs (At3g07070 and At4g02630) interacted with BIK1 (Figure 5). The 17 RLCKs tested in Figure 4 were fused with the C-terminal fragment of YFP and co-infiltrated into *Nicotiana benthamiana* (tabacco) leaves, along with AtBIK1 fused to the N-terminal fragment of YFP. After 48 h of infiltration, tobacco leaves were observed under a confocal fluorescence microscope. *SOS2*-YFPn and *SOS3*-YFPc were used as positive controls. YFPn and YFPc were used as negative controls.

## 4. Discussion

Receptor kinases have been thoroughly studied in animal and plant systems and play a proven role in many signal transduction pathways. For example, early studies demonstrated that when BRI1-CD was incubated with [γ-^32^P]-ATP, phosphorylation of threonine and serine residues was readily detected [36]. Also, staining with Pro-Q Diamond phosphoprotein stain and immunoblotting with anti-phosphothreonine antibodies demonstrated that the CDs of LRR-RLKs, including Arabidopsis BRI1 and BAK1, are highly autophosphorylated as purified from *E. coli*, and many of these sites have now been identified by mass spectrometry and phosphospecific peptide antibodies [24]. The occurrence of tyrosine phosphorylation was confirmed for BRI1 both in vitro and in vivo. In particular, plants use immune receptors on the cell surface and inside cells to recognize and defend against pathogens. The receptor-like protein (RLP) super family functions as cell surface receptors in plant disease resistance, and they function as part of the PRR or PRR complex [37]. Interestingly, RLPs are involved in the direct immune response of plants and are involved in the PTI response or the ETI response to resist effectors generated from pathogens. PEPR1 is one of the LRR-RLK families present in plants, and it forms a complex with BAK1, recognizes the Pep1 peptide, and activates the defense system [38,39,40]. In addition, although the phosphorylation of threonine and serine residues has been mainly studied in plants, studies on the phosphorylation of tyrosine residues in the LRR-RLK of plants have been reported. For example, brassinosteroid insensitive 1 (BRI1), which recognizes the plant hormone brassinosteroid, undergoes a tyrosine phosphorylation at the Y831 site. When this site was substituted with phenylalanine or aspartic acid, significant molecular and phenomics data were reported [24,41], and studies on HAESA, ERL1, PSKR1, and FLS2 in plants also reported protein structural changes caused by the phosphorylation of tyrosine residues in various LRR-RLKs. In addition, studies on tyrosine phosphorylation in plants have been continuously reported, such as the important role of the phosphorylation of tyrosine residues not only in LRR-RLK but also in the mechanism involved in stomatal opening and closing [42,43]. 

As mentioned in the introduction, PEPR1 and PEPR2 recognized the pep1 peptide and activated the plant’s innate immune response. Understanding the immune system of plants is becoming increasingly important. In this study, we investigated PEPR1 and PEPR2 as members of the LRR-RLK family and aimed to understand the pep1 peptide-triggered immune response. For this, we examined the tyrosine autophosphorylation site of PEPR1. As a result, we confirmed that Tyr-995 is a critical autophosphorylation site in PEPR1-CD. The tyrosine phosphorylation intensity of the Y995F mutant was reduced to half the level, compared to normal PEPR1-CD and other mutants. Since other mutants, including PEPR2-CD mutants, showed no change in tyrosine autophosphorylation intensity, it can be inferred that Tyr-995 in PEPR1-CD plays a key role in the pep1-triggered immune response. Moreover, the threonine autophosphorylation intensity of the Y995F mutant remained almost unchanged. Therefore, tyrosine autophosphorylation, particularly at the Tyr-995 site, is essential for PEPR1 function, especially its kinase catalytic activity.

Based on this, we investigated how Tyr-995 functions in *Arabidopsis thaliana* under pep1-treated conditions. To do so, we generated transgenic plants to study PEPR1 function. When PEPR1, which is localized at the cell membrane of *Arabidopsis*, recognized the pep1 peptide, the plant’s defense system was activated, leading to a near cessation of root growth, particularly inhibiting primary root growth and root hair formation [44]. This suggested that a normal defense signaling cascade was transmitted to downstream components. The functional significance of Tyr-995 autophosphorylation was confirmed based on root growth under pep1-treated conditions. Considering that no significant differences were observed among plants under normal growth conditions without pep1 treatment, knock-out and transgenic lines did not appear to affect growth under normal conditions. However, when 1 μM Pep1 was treated, root growth was inhibited in Col-0, whereas root length remained similar to that of the *pepr1/2* knock-out plant in PEPR1(Y995F)-Flag and PEPR1(Y995D)-Flag transgenic plants. This suggests that these plants failed to transmit the pep1 signal. In *pepr1/2* mutants, pep1 was naturally not recognized due to the complete loss of its receptor. However, in the two transgenic lines, tyrosine phosphorylation was specifically blocked, leading to a similar response as *pepr1/2* mutants. Therefore, phosphorylation at the Tyr-995 site of PEPR1 plays a crucial role in the pep1-triggered signaling cascade.

As shown in Figure 2, since we demonstrated that the Tyr-995 site played a key role in pep1-triggered defense signaling at the phenotypic level, we conducted qRT-PCR to confirm this through gene expression analysis. PROPEP1 is a precursor of Pep1, and its expression level significantly increased when the defense mechanism was activated by Pep1 [14]. Mitogen-activated protein kinase 3 (MPK3) is involved in the plant defense system alongside MPK6 and has been reported to transmit phosphorylation signals in the cytosol [45]. WRKY33 is a transcription factor involved in plant defense by receiving signals from MPK3 and MPK6 [46,47]. Additionally, RBOHD generates reactive oxygen species (ROS) as part of the plant defense response by receiving signals from the PEPR1–Pep1 complex [16,39,48]. These genes were normally regulated in Col-0 and PEPR1-Flag transgenic plants. However, in PEPR1(Y995F)-Flag and PEPR1(Y995D)-Flag, their expressions were not regulated, similar to *pepr1/2* double knock-out plants. This provides both phenotypic and gene expression evidence that autophosphorylation at Tyr-995 of PEPR1 is essential for PEPR1 function and the normal transmission of the pep1-triggered signaling cascade.

Most LRR-RLKs form complexes with co-receptors, such as PEPR1-BAK1 [40], and/or RLCKs, such as PEPR1-BIK1 [49,50], to function. Given that no RLCKs interacting with PEPR1 were identified except for BIK1, which interacted with FLS2, EFR, and PEPR1, we sought to identify additional RLCKs that interact with PEPR1. As shown in Figs. S1 and 4, the expressions of 20 RLCKs we analyzed were regulated by Pep1 treatment. Among these, 18 RLCKs appeared to be more closely involved in pep1 signaling, as their expressions were not only regulated in Col-0 and PEPR1-Flag transgenic plants but were also absent in *pepr1/2* double knock-out plants, PEPR1(Y995F)-Flag, and PEPR1(Y995D)-Flag transgenic plants.

The nine RLCKs up-regulated by pep1 were primarily involved in plant immune responses or stress regulation, suggesting their possible involvement in the pep1 signaling cascade. For example, *At5g10520* (ROP-binding protein kinase 1, RBK1) was reported to function in basal penetration resistance [51], while *At2g11520* (Calmodulin-binding receptor-like cytoplasmic kinase 3, CRCK3) plays a role in pathogen resistance [52,53]. Meanwhile, the nine RLCKs down-regulated by pep1 served various functions. Some, such as *At2g28250* (novel cysteine-rich receptor kinase, NCRK) and *At5g59010* (brassinosteroid-signaling kinase 5, BSK5), are involved in plant stress regulation or immunity. Others, like *At3g07070* (PBS1-like 26, PBL26) and *At5g46570* (brassinosteroid-signaling kinase 2, BSK2), function in plant growth and development [54,55,56,57]. This indicates that the 18 RLCKs regulated by pep1 may be components of the PEPR1 complex or interact with it directly or indirectly. Through positive and negative feedback mechanisms, they may collectively balance plant immune responses, stress regulation, growth, and development within the framework of growth–defense tradeoffs in plants.

As a final step, we focused on identifying physical interactions between PEPR1 and RLCKs. However, no direct interaction between PEPR1 and RLCKs was discovered. Nonetheless, since PEPR1 has a known direct interaction with BIK1 [49,58], we attempted to identify indirect interactions through BIK1. As a result, two RLCKs (*At3g07070* and *At4g02630*) were found to interact with BIK1. Given that the expressions of these RLCKs were regulated by pep1 treatment and that they interacted with BIK1, it can be inferred that *At3g07070* and *At4g02630* either interacted indirectly with PEPR1 via BIK1 or played a role in pep1 signaling. As previously mentioned, *At3g07070* (PBL26) is involved in plant growth and development, whereas the function of *At4g02630* remains unclear. Since both RLCKs were down-regulated by pep1 treatment and considering the known function of *At3g07070*, they may act as negative regulators of the pep1 signaling pathway.

## 5. Conclusions

In summary, we identified Tyr-995 as a major autophosphorylation site in PEPR1. Substitution of Tyr-995 with phenylalanine markedly reduced tyrosine autophosphorylation compared to wild-type PEPR1 and other mutants. Subsequently, we generated a phospho-specific antibody against pY995 and confirmed that Tyr-995 is an autophosphorylation site in vitro. Furthermore, transgenic plants expressing mutant forms of PEPR1 were used to assess the functional significance of Tyr-995 in pep1-triggered responses. The results from root length measurements and downstream gene expression analyses demonstrated that phosphorylation at Tyr-995 is critical for effective signal transduction. Mutants harboring Y995F and Y995D substitutions failed to transmit pep1 signals to downstream components, indicating the importance of this phosphorylation site in defense signaling. To identify RLCKs involved in pep1-triggered signaling, gene expression analyses of RLCKs under pep1-treated conditions were conducted. Among these, At3g07070 and At5g46570, which interact with BIK1, emerged as potential candidate RLCKs within the PEPR1 complex at the plasma membrane of *Arabidopsis thaliana*.

## Data Availability

The original contributions presented in this study are included in the article/Supplementary Material. Further inquiries can be directed to the corresponding author.

## Supplementary Materials

The following supporting information can be downloaded at: https://www.mdpi.com/article/10.3390/plants14101515/s1, Figure S1. Expression analysis of RLCK genes in Col-0 after pep1 treatment. Figure S2. AtPEPR1 interacts with AtBIK1 at plasma membrane. Table S1. Primers list.
