# Supplementary material for: Role of Tyrosine Phosphorylation in PEP1 Receptor 1(PEPR1) in Arabidopsis thaliana
Source: Plants (Basel). 2025 May 19;14(10):1515. doi: 10.3390/plants14101515 (PMC12115080; doi:10.3390/plants14101515)
Supplement: Supplementary file 1 [file plants-14-01515-s001.zip › plants-3628378-supplementary.pdf]

plants

MDPI

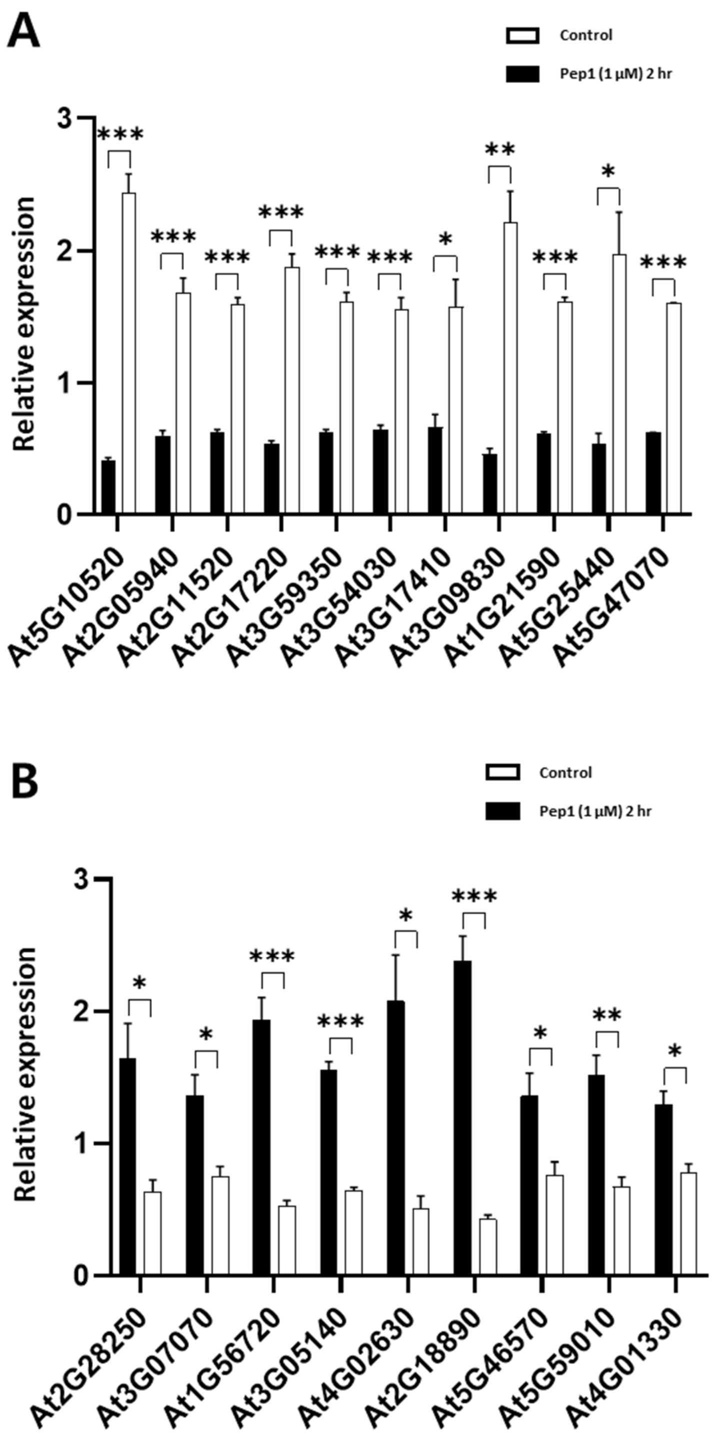

Figure S1. Expression analysis of RLCK genes in Col-0 after pep1 treatment. The effects of pep1 on the expression of 78 RLCKs in Col-0. Seedlings of *Arabidopsis* was cultured in half-strength MS medium for 10 days and treated with pep1 peptide for 2hr. The relative expression was normalized to that of ACTIN. Three independent biological repeats were conducted. Error bar represent SE. (**A**) RLCKs up-regulated by pep1. (**B**) RLCKs down-regulated by pep1.

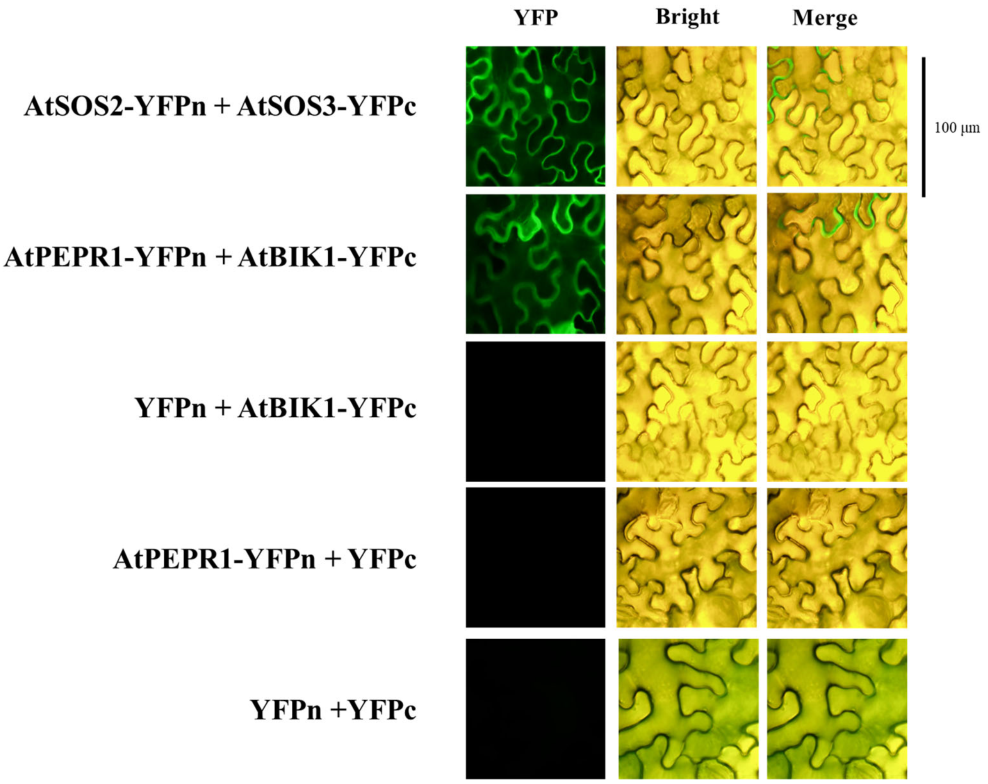

Figure S2. AtPEPR1 interacts with AtBIK1 at plasma membrane. BiFC assay demonstrated that interaction between PEPR1 and BIK1 in tobacco (*Nicotiana benthamiana*) leaves. Genes fused with the N-terminal or C-terminal fragment of YFP(YFPn or YFPc) were co-introduced into tobacco leaves. AtSOS2-YFPn and AtSOS3-YFPc were used positive control. Each empty vector complex was used negative control.

Table S1. Primers list.

Oligo Name	Sequence
PROPEP1 (At5g64990) qF	GAA CTT CGA AAC AGC CGA AG
PROPEP1 (At5g64990) qR	ACT CGT CAC AAC GAC CTC CT
AtMPK3 (At3g45640) qF	GAG TTC GAA CAA CAG CCT CTG
AtMPK3 (At3g45640) qR	CCG TAT GTT GGA TTG AGT GCT
AtWRKY33 (At2g38470) qF	AAA TAC GGC CAG AAA GTC GTT
AtWRKY33 (At2g38470) qR	TGT TTC CCT TCG TAG GTT GTG
AtRBOHD (At5g47910) qF	GAA AGG AGT GGA AGG ATG GAC
AtRBOHD (At5g47910) qR	GTG TAC CAA AAG GCG TTG AAA
At5g10520 qF	TGG TGA GAC GGT GGC GAT TA
At5g10520 qR	GAT CAC TGC TGA AAC CGC GA
At2g05940 qF	CAC TTA CAC GGT GCC AAA CA
At2g05940 qR	GAC GAC CGG GGA TGA TTA CT
At2g11520 qF	AGC GAC TTC CTG ATG AGA GG
At2g11520 qR	TCA TCT ACT CGT TCC CTC GC
At2g17220 qF	GCA GCT CCA GAG TAT GTT GC
At2g17220 qR	AGG ACG AGT AGG ATC AAG CG
At3g17410 qF	GTC ACC ATC AAA GGG CAG AT
At3g17410 qR	GAA CCC TCA CCA ATC AAG GA
At3g09830 qF	AAG GCA CGT CCC AAG ATG AGT G
At3g09830 qR	TGT GGG CTG CCA TTT CCT GAA G

At1g21590 qF	ACT AGG CCA ACG ATG GGA ATG GTC
At1g21590 qR	CCT CCG CAA TTT CTC GTC CTT GAG
At5g25440 qF	ACC CAC CTC CCG AAT ACA TAC
At5g25440 qR	TTC TCG CTT TGC TGA GAT TGT
At5g47070 qF	GAA AGC TTG TGA TCG GTG AAG
At5g47070 qR	TTG CTT GTG ACC CTG TAA ACC
A2g28250 qF	CGG AAG GAA ACC GAT ACA GA
A2g28250 qR	GCA TCT CTT CTT CCG CAA AC
At3g07070 qF	GCT CAA ACA TTC AGC TTC AGG
At3g07070 qR	AAT TGC TTC ACT GCC ACA ATC
At1g56720 qF	AAA AAG CCA TGT GAC AAC TCG
At1g56720 qR	TCT CGT CCT GTG ATT GCT TCT
At3g05140 qF	CGA TTT CCA ATC ATC TCT CCA
At3g05140 qR	ACA GCG ATT AGC TTT CCT TCC
At4g02630 qF	TGT GAT GGG TAC GTT TGG GTA
At4g02630 qR	ATC CAC AGG ACT TCT CCC TGA
At2g18890 qF	TGG GCA CTT AGC ACC AGA GTA
At2g18890 qR	TGT AGG CTT TGG TGA GAA GCA
At5g46570 qF	CCA CCT GAG TTT TTA CGG ACA
At5g46570 qR	CAT GGC TAG GTG GAA TGT GTT
At5g59010 qF	AGC TGC TTC TCC AAA GGA AAC
At5g59010 qR	CTT GGA ACG AGA GCT CAT TTG
At4g01330 qF	TCA TGG GGA TGT TGG TGA TAA
At4g01330 qR	ACT TTC GGT TCA AGA CCC TCA
